# Long noncoding RNA MALAT1 potentiates growth and inhibits senescence by antagonizing ABI3BP in gallbladder cancer cells

**DOI:** 10.1186/s13046-019-1237-5

**Published:** 2019-06-07

**Authors:** Nan Lin, Zhicheng Yao, Mingxing Xu, Jingyao Chen, Yi Lu, Lin Yuan, Shuqin Zhou, Xiaoguang Zou, Ruiyun Xu

**Affiliations:** 1 Department of Hepatobiliary Surgery, The Third Affiliated Hospital, Sun Yat-sen University, No. 600, Tianhe Road, Tianhe District, Guangzhou, 510630 Guangdong Province People’s Republic of China; 2 Department of General Surgery, The Third Affiliated Hospital, Sun Yat-Sen University, Guangzhou, 510630 People’s Republic of China; 3 Department of Anesthesiology, Zhujiang Hospital of Southern Medical University, Guangzhou, 510280 People’s Republic of China; 4 Department of Cardiology, Kashi Hospital Affiliated to Sun Yat-sen University, Kashi, 844000 People’s Republic of China

**Keywords:** Metastasis associated lung adenocarcinoma transcript 1, ABI family member 3 binding protein, Gallbladder cancer, Enhancer of zeste homolog 2, Histone, Methylation, Growth, Senescence

## Abstract

**Background:**

Gallbladder cancer (GBC) is the most malignant cancer occurring in the biliary tract cancer featured with undesirable prognosis, in which most patients die within a year of cholecystectomy. Long noncoding RNAs (lncRNAs) function as critical regulators of multiple stages of cancers. Herein, the mechanism of lncRNA metastasis associated lung adenocarcinoma transcript 1 (MALAT1) in GBC is investigated.

**Methods:**

Microarray-based analysis initially provided data suggesting that the expression of MALAT1 was up-regulated while that of the ABI family member 3 binding protein (ABI3BP) was down-regulated in GBC tissues and cell lines. Kaplan-Meier method was then adopted to analyze the relationship between the MALAT1 expression and overall survival and disease-free survival of patients with GBC. A set of in vitro and in vivo experiments were conducted by transducing ABI3BP-vector or sh-MALAT1 into GBC cells.

**Results:**

The results confirmed that the cancer prevention effects triggered by restored ABI3BP and depleted MALAT1 as evidenced by suppressed cell growth and enhanced cell senescence. MALAT1 was observed to down-regulate ABI3BP expression through recruitment of the enhancer of zeste homolog 2 (EZH2) to the ABI3BP promoter region while the silencing of MALAT1 or suppression of H3K27 methylation was observed to promote the expression of ABI3BP. Furthermore, GBC patients with high expression of MALAT1 indicated poor prognosis.

**Conclusion:**

The current study clarifies that MALAT1 silencing and ABI3BP elevation impede the GBC development through the H3K27 methylation suppression induced by EZH2, highlighting a promising competitive paradigm for therapeutic approaches of GBC.

## Background

Gallbladder cancer (GBC) is a malignant cancer occurring in the biliary tract and has been highlighted to be frequent occurrence in developing countries, with adverse outcomes of the treatment due to the undesirable prognosis and late diagnosis [1]. Recent evidence has ranked GBC as the 7th most frequently occurring gastrointestinal cancer, with approximately 2.5 in 100,000 persons affected, with a survival time of less than 1 year regardless of adjuvant therapy of standard chemotherapy [2]. Existing literature has emphasized that the genomic scenario and biomarker-oriented trials in clinical practice represent the future of GBC treatment [3]. Thus, it is of great significance to uncover the mechanism of GBC on the molecular level to facilitate the evolution of novel biomarkers and better therapeutic modalities.

Accumulating evidence has demonstrated that long non-coding RNAs (lncRNAs), such as lncRNA KIAA0125, lncRNA GCASPC and lncRNA H19, serve as key regulators in the biological functions of GBC cells [4–6]. Metastasis associated lung adenocarcinoma transcript 1 (MALAT1) represents a novel lncRNA localized in human chromosome 11q13, which is expressed in abundance in various mammalian species, from a physiological and pathophysiological perspective [7]. MALAT1 has been implicated in colorectal cancer metastasis and bladder cancer cell migration [8, 9], highlighting its ability to participate in in carcinogenesis. Crucially, the correlation between MALAT1 and GBC has been speculated to work with the extracellular signal-regulated kinase/mitogen-activated protein kinase signaling pathway, but the underlying molecular mechanism remains poorly understood [10]. ABI3BP is a gene that encodes extracellular matrix proteins linked with proliferation, differentiation and cellular senescence [11]. A previous study demonstrated the ability of ABI3BP to serve as a regulator of cardiac progenitor cell proliferation and differentiation [12]. ABI3BP has been suggested to have tumor suppressive abilities in thyroid carcinoma [13]. Hence, it was inferred that ABI3BP may also possess the ability to mediate the pathogenesis and/or progression of GBC. DNA methylation represents as epigenetic mechanism responsible for gene expression regulation [14]. The correlation between DNA and histone lysine methylation systems and its influence on normal chromatin functions in vivo has been reported [15]. Evidence of the suppressive effect of ABI3BP on carcinogenesis relates to the instable chromosome [16]. The aim of the current study was to investigate the mechanism by which MALAT1 and ABI3BP influence GBC, in an attempt to identify a novel diagnostic and prognostic biomarker for better understanding the pathogenesis and treatment of GBC.

## Materials and methods

### Ethics statement

The study conducted with the approval of the Institutional Review Board of The Third Affiliated Hospital, Sun Yat-sen University and Zhujiang Hospital of Southern Medical University. Written informed consent was obtained from each participant. The animal protocol and experiment procedures performed with the approval of the Institutional Animal Care and Use Committee of The Third Affiliated Hospital, Sun Yat-sen University and Zhujiang Hospital of Southern Medical University.

### Study subjects

A total of 48 patients with GBC were enrolled in the study between June 2016 and June 2017. Of the enrolled participants, 26 were males while 22 were females (mean age: 43.06 ± 8.92 years, ranging from 26 to 61 years). All enrolled patients underwent cholecystectomy surgical procedures. Additional 16 patients, consisting of 10 males and 6 females (mean age: 44.81 ± 7.72 years, ranging from 30 to 50 years) diagnosed with cholecystitis were also enrolled. None of the included GBC patients were treated with antitumor therapy prior to the surgery. All the GBC patients were confirmed by the pathological diagnosis and could be treated with radical mastectomy based on imaging examination. GBC was further confirmed by pathological diagnosis. Patients with distant metastasis, and cachexiawere excluded from the study. The control group and the case group were matched on the basis of age and gender at a ratio of 1: 3. The patients with the control group were patients with the cholecystolithiasis complicated with the chronic cholecystitis, and the specimen were obtained by cholecystectomy. The participants in the control group were confirmed to be suffering from cholecystolithiasis complicated with the chronic cholecystitis and agreed to participate in the study. The basic information of all patients was collected, with each patient followed-up in order to gain a detailed understanding of the situation and clinical outcome post treatment. The follow-up period commenced post-surgery until the end of December 2018 for a period of 3–30 months. The Kaplan-Meier method was employed in order to evaluate the relationship between the expression of MALAT1 and total survival (OS) and disease-free survival (DFS).

### Immunohistochemistry

Paraffin-embedded sections were dewaxed to water, dehydrated using an ascending series of ethanol and washed under running water for 2 min. Then, 3% H_2_O_2_ was employed in order to immerse the sections at room temperature for 20 min, followed by antigen retrieval in a water bath. After being blocked with normal goat serum (C-0005, Shanghai Haoran Biological Technology Co., Ltd., Shanghai, China) at room temperature for 20 min, the samples were incubated with the primary rabbit anti-human ABI3BP antibody (ab187899, 1: 200, Abcam Inc., Cambridge, MA, USA) at 4 °C overnight. After phosphate buffer saline (PBS) washing, the sections were probed with secondary antibody, biotin-labeled goat anti-rabbit antibody to immunoglobulin G (IgG; 1: 1000, ab6785, Abcam Inc., Cambridge, MA, USA) at 37 °C for 20 min. The horseradish peroxidase (HRP)-conjugated streptavidin working solution (0343-10,000 U, Imunbio co., Ltd., Beijing, China) was added and probed with the sample at 37 °C for 20 min, followed by diaminobenzidine (DAB; ST033, Whiga Biotech Co., Ltd., Guangzhou, Guangdong, China) development and hematoxylin (PT001, Bogoo Biotech Co., Ltd., Shanghai, China) staining for 1 min. The sections were returned to blue using 1% ammonia, dehydrated with gradient alcohol, cleared with xylene and mounted using neutral resin. The section was subsequently analyzed and photographed under an optical microscope. Five fields in high power visual were selected on a random basis with 100 cells in each field. The experiment was repeated 3 times.

### Primary culture

The internal wall of gallbladder was washed 3–4 times with Dulbecco’s Modified Eagle Medium (DMEM) in order to remove the mucus from mucosa, followed by the addition of 0.2% collagenase IV. The opening of the gallbladder was clamped, followed by detachment at 37 °C for 20 min. The gallbladder was then vertically cut and tiled on a sterile plate, with one side fixed with a tissue forceps. The gallbladder mucosa was scraped in a cautious manner using a blade. The cell suspension was harvested and filtered through a 100-mesh net, followed by centrifugation at 1000 rpm for 10 min. After the precipitate had been washed twice with DMEM, the cell suspension was seeded into the 24-well plate at a density of 1 × 10^5^ cells/mL and cultured at 37 °C with 5% CO_2_ and saturated humidity. The medium was renewed 24 h later and half of medium was renewed every 2 days afterwards. On the 3rd day, immunohistochemical staining was conducted in a conventional manner using the rabbit monoclonal antibody to gallbladder epithelial cell marker keratin CK19 (1: 200–1: 500, ab52625) as the primary antibody while HRP-conjugated goat anti-rabbit IgG was employed as the secondary antibody, followed by DAB development and hematoxylin counterstaining. Cell coloring and cell purity were subsequently analyzed. The brown cells marked by the CK19 antibody were considered to be the gallbladder epithelial cells. When cell confluence reached approximately 80%, the cells were detached by trypsin and passaged accordingly. The cells at the exponential growth phase were selected for subsequent experimentation.

### Cell treatment

GBC cell lines, GBC-SD, SGC-996, NOZ and OCUG1 were purchased from American Type Culture Collection (ATCC, Manassas, VA, USA). The primarily cultured human gallbladder epithelial cells (HGBECs) were served as the control. Cells were cultured with Roswell Park Memorial Institute (RPMI) 1640 medium (Gibco, Carlsbad, CA, USA) containing 10% fetal bovine serum (FBS; Gibco, Carlsbad, CA, USA), 10 μg/mL streptomycin and 100 U/mL penicillin in the incubator (Thermo Fisher Scientific Inc., CA, USA) at 37 °C with 5% CO_2_. The expression of MALAT1 in GBC-SD, SGC-996, NOZ, OCUG1 cells and HGBECs was determined by means of reverse transcription quantitative polymerase chain reaction (RT-qPCR). The cells at the exponential growth phase were detached and seeded in a 6-well plate at a density of 1 × 10^5^ cells/well and cultured for 24 h (h). When cell confluence had reached approximately 75%, transfection was initiated in accordance with the instructions of Lipofectamine 2000 (Invitrogen Inc., Carlsbad, CA, USA). The lentivirus particles containing the specific short hairpin RNAs (shRNAs) for negative control (sh-NC, 5′-GGGUGAACUCACGUCAGAA-3′), MALAT1–1 (sh-MALAT1–1, 5′-CACAGGGAAAGCGAGUGGUUGGU-3′), MALAT1–2 (sh-MALAT1–2, 5′-GACAGGUAUCUCUUCGUUAUC-3′) and MALAT1–3 (sh-MALAT1–3, 5′-GACCUUGAAAUCCAUGACG-3′) were transduced into cells, respectively. After 48 h, the interference efficiency was detected by RT-qPCR. The ABI3BP-vector plasmid (50 ng/mL) and vector-NC plasmid (50 ng/mL) purchased from GenePharma Co., Ltd. (Shanghai, China) were respectively transfected into cells. The cells were then grouped based on their treatment with dimethyl sulfoxide (DMSO), GSK343 (enhancer of zeste homolog 2 [EZH2] inhibitor) or ubiquitously transcribed tetratricopeptide repeat on chromosome X (UTX) (histone demethylase).

### RNA isolation and quantitation

Total RNA was extracted using the Trizol regent (15,596,026, Invitrogen, Carlsbad, CA, USA). RNA was reversely transcribed into cDNA in accordance with the instructions of the PrimeScript RT reagent Kit (RR047A, Takara, Tokyo, Japan). Next, the cDNA was subjected to qPCR using the Fast SYBR Green PCR kit (Applied Biosystems, Waltham, MA, USA) on the ABI PRISM 7300 RT-PCR system (Applied Biosystems, Waltham, MA, USA). Three duplicates were set for each group. Glyceraldehyde 3-phosphate dehydrogenase (GAPDH) was regarded as the internal control. The fold changes of MALAT1 were calculated by the relative quantification (2^-ΔΔCt^ method). The primer sequences for RT-qPCR are illustrated in Table 1.

**Table 1:** Primer sequences for reverse transcription quantitative polymerase chain reaction

Gene	Primer sequences
MALAT1	F: 5′-AAAGCAAGGTCTCCCCACAAG-3’
	R: 5′-GGTCTGTGCTAGATCAAAGGCA-3’
GAPDH	F: 5′-GTGGACCTGACCTGCCGTCT-3’
	R: 5′-GGAGGAGTGGGTGTCGCTGT-3’
ABI3BP	F: 5′-CCATCTGGACTGAAAGACCCTT-3’
	R: 5′-CCACAAACTGGCAGTGATCTTC-3’

Note: MALAT1, metastasis associated lung adenocarcinoma transcript 1; GAPDH, glyceraldehyde-3-phosphate dehydrogenase; ABI3BP, ABI family member 3 binding protein

### Western blot analysis

The cells were detached by trypsin, collected and lysed using an enhanced radioimmunoprecipitation assay (RIPA) lysis buffer (Boster Biological Technology Co., Ltd., Wuhan, Hubei, China) containing protease inhibitor. The protein concentration was determined using a bicinchoninic acid (BCA) protein quantification kit (Boster Biological Technology Co., Ltd., Wuhan, Hubei, China). The protein was separated with 10% sodium dodecyl sulfate-polyacrylamide gel electrophoresis (SDS-PAGE). The separated proteins were transferred onto a polyvinylidene fluoride (PVDF) membrane, which was then blocked using 5% bovine serum albumin (BSA) for 2 h to prevent non-specific binding. The membrane was probed with diluted (1: 500) primary antibodies purchased from Abcam Inc., (Cambridge, CA, USA) at 4 °C overnight. The primary antibodies used in the study included rabbit antibodies to proliferating cell nuclear antigen (PCNA; ab18197), Ki67 (ab16667), trimethylation of lysine 27 on histone H3 protein subunit (H3K27me3; ab222481) and β-actin (ab8227). After a series of washes, the membrane was then incubated with the secondary antibody, HRP-conjugated goat anti-rabbit antibody (1: 2000, ab205719, Abcam Inc., Cambridge, CA, USA) at room temperature for 1 h. The membrane was developed by the enhanced chemiluminescence (EMD Millipore, Temecula, CA, USA). The gray value was quantified and analyzed using Image J software. β-actin was regarded as the internal control. The experiment was repeated 3 times.

### 5-ethynyl-2′-deoxyuridine (EdU) staining

The cells were seeded into the 24-well plate, with three duplicates set for each group. EdU solution was added in order to reach a final concentration of 10 μmol/L followed by incubation for 2 h. The cells were then fixed by PBS containing 4% paraformaldehyde at room temperature. The cells were then washed with PBS supplemented with 3% BSA and incubated with 0.5% Triton-100 at room temperature for 20 min, followed by two additional PBS washes. The cells in each well were stained with 100 μL staining solution for 30 min at room temperature under conditions void of light. The nucleus was stained with 4′,6-diamidino-2-phenylindole (DAPI) for 5 min. Next, 6–10 visual fields were randomly observed and photographed under a fluorescence microscope (FM-600, Shanghai Pudan Optical Instrument Co., Ltd., Shanghai, China), with the number of positive cells subsequently recorded. EdU labeling (%) = the number of positive cells/(the number of positive cells + the number of negative cells) × 100%. The experiment was repeated 3 times.

### Scratch test

A minimum of 5 horizontal lines were drawn in an even fashion at the bottom of each well of the 6-well plate at 0.5–1 cm intervals. Cells were seeded at a density of 5 × 10^5^ cells/well and cultured in medium supplemented with 10% FBS overnight. The scratches were perpendicular to the horizontal lines using a 10 μL sterile pipette. The distance between the two sides of the scratch was measured at the 0th h and 24th h, after which images were acquired under an inverted microscope.

### Transwell assay

The Transwell apical chamber was coated with Matrigel (BD Biosciences, FL, NJ, USA) and permitted to stand at 37 °C for 30 min to polymerize Matrigel. The basement membrane of the Transwell was hydrated before use. The cells were cultured in serum-free medium for 12 h and re-suspended with serum-free medium into a density of 1 × 10^5^/mL. The basolateral chambers were added with medium containing 10% FBS. Cell suspension (100 μL) was then added to the Transwell apical chamber for a 24-h period of incubation at 37 °C. The cells that failed to invade the Matrigel were removed using a cotton swab. The cells were fixed in 100% methanol and stained with 1% toluidine blue (Sigma, St. Louis, MO, USA). Five fields were randomly selected with the number of invaded cells counted artificially under the guidance of an inverted optical microscope (CarlZeiss Meditec AG Inc., Oberkochen, Germany). The experiment was repeated 3 times.

### Flow cytometry

The GBC cells were seeded into the 6-well plate (1 × 10^6^ cells/well) and transfected with siRNA. After 48 h, the cells were stained using both fluorescein isothiocyanate (FITC)-Annexin V and propidium iodide (PI) (BD Biosciences, FL, NJ, USA) as per the instructions of the Annexin V-FITC/PI apoptosis detection kit (BD Biosciences, FL, NJ, USA). Cell apoptosis was detected by flow cytometry (FACS, BD Biosciences, FL, NJ, USA). The experiment was repeated 3 times.

### Senescence-associated-β-galactosidase (SA-β-gal) staining

The cells were cultured in a 6-well plate. After 24 h, the culture medium was aspirated after which the cells were washed with PBS and fixed using 1 mL SA-β-gal fixative solution for each well for 20 min at room temperature. After the removal of the fixative solution, the cells were washed 3 times with PBS (3 min each time). Next, 1 mL of staining working solution was added into each well. The 6-well plate was then sealed by the sealing film and incubated at 37 °C overnight. Cell-counting was subsequently performed under an ordinary microscope. The experiment was repeated 3 times.

### Fluorescence in situ hybridization (FISH)

The subcellular localization of the MALAT1 cells was identified through the application of FISH as per the instructions of the Ribo™ lncRNA FISH Probe Mix (Red) Kit (Ribobio Co., Ltd., Guangzhou, Guangdong, China). The coverslip was placed onto the well of the 6-well plate, where HGBECs were seeded at a density of 1 × 10^5^ cells/well. When the cell confluence reached approximately 80% on the next day, the coverslip was washed with PBS, fixed with 1 mL 4% paraformaldehyde, and treated with proteinase K (2 μg/mL), glycine and acetylation reagent. The cells were then prehybridized with 250 μL prehybridization solution at 42 °C for 1 h and then hybridized with 250 μL hybridization solution containing probe (300 ng/mL) at 42 °C overnight. After an additional 3 phosphate buffered saline with Tween-20 (PBST) washes, the nucleus was then stained with PBST-diluted DAPI (1: 800) for 5 min. The cells were then washed 3 times with PBST (3 min each time), and mounted with anti-fluorescence quenching agent. The stained cells were analyzed and photographed under a fluorescence microscope (Olympus Corp., Tokyo, Japan) with five visual fields randomly selected.

### RNA immunoprecipitation (RIP)

The binding of MALAT1 to EZH2 protein was detected by the RIP kit (Millipore, Temecula, CA, USA). The cells were washed with the pre-cooled PBS, and lysed with the radioimmunoprecipitation assay (RIPA) lysis buffer (P0013B, Beyotime Biotechnology Co., Ltd., Beijing, China) in the ice-bath for 5 min. The supernatant was collected after a centrifugation at 14000 rpm for 10 min at 4 °C. Partial cell extract was used as Input while the other part was incubated with rabbit antibody to EZH2 (ab19125, 1: 100, Abcam Inc., Cambridge, MA, USA) at room temperature for 30 min for co-precipitation, while the rabbit anti-human IgG (ab109489, 1: 100, Abcam Inc., Cambridge, MA, USA) was regarded as the negative control. Total RNA was extracted from the sample and Input after protease K detachment. The MALAT1 content was quantified by RT-qPCR means. The experiment was repeated 3 times.

### RNA pull-down assay

The GBC cells were transfected with wild type (WT) biotinylated MALAT1 (50 nM) and mutant (MUT) biotinylated MALAT1 (50 nM) in a respective manner. Next, 48 h later, the cells were collected, and washed with PBS. The specific cell lysis (Ambion, Austin, TX, USA) was added for a 10-min period of incubation. A part of the cell lysate was then sub-packed accordingly. The remaining lysate was incubated with M-280 streptavidin beads (Sigma, St. Louis, MO, USA) pre-coated with RNase-free yeast tRNA (Sigma, St. Louis, MO, USA) at 4 °C for 3 h. The sample was then washed twice with the pre-cooled lysis buffer, 3 times with low-salt buffer solution and once with the high-salt buffer solution. The total protein was extracted. The expression of EZH2 was determined by Western blot analysis.

### Chromatin immunoprecipitation (ChIP)

The assay was implemented in accordance with the provided instructions of the ChIP kit (Millipore, Temecula, CA, USA). When the cell confluence reached 70–80%, the cells were fixed in 1% formaldehyde at room temperature for 10 min in order to cross-link the cellular DNA with protein. An ultrasonic breaker was set to 2 s per ultrasonic cycle with 5-s intervals with 15 cycles to break the chromatin at 120 w. The supernatant was collected after a centrifugation at 4 °C at 13000 rpm and sub-packed into 3 tubes, each of which was probed with NC antibody RNA polymerase II, NC antibody normal human IgG and rabbit antibody to EZH2 (1: 100, ab19125, Abcam Inc., Cambridge, MA, USA) at 4 °C overnight, respectively. The endogenous DNA-protein complex was precipitated using Protein Agarose/Sepharose, followed by transient centrifugation. After the removal of the supernatant was discarded, the non-specific complex was washed and de-crosslinked at 65 °C overnight. DNA fragments were extracted and purified by phenol/chloroform. RT-qPCR was then performed to determine the expression of the ABI3BP promoter. The experiment was repeated 3 times.

### Xenograft tumor in nude mice

A total of 36 healthy nude mice (aged 6–8 weeks, Institute of Material Medical, Chinese Academy of Medical Sciences, Beijing China) were separately housed in specific pathogen free (SPF)-level animal room with humidity of 60–65% at 22–25 °C under a 12-h light and 12-h darkness cycle with free access to food and drinking water. The experiment was initiated after a 1-week adaptive feeding period. The health status of each mouse was determined prior to the experiment. The mice were divided into 6 groups with 6 mice placed in each group. The mice were injected with adenovirus vectors of ABI3BP-vector, sh-MALAT1, sh-EZH2, sh-MALAT1 + sh-ABI3BP, vector-NC and sh-NC via tail, respectively (1 × 10^9^ pfu/100 μL, twice a week, for 6 weeks). All mice were subsequently euthanized after the tumors had been resected. The size and weight of tumors were measured. The expression of Ki67 and PCNA, the capabilities of migration and invasion and the SA-β-gal activity were detected. The experiment was repeated 3 times.

### Statistical analysis

All experimental data was processed by SPSS 21.0 statistical software (IBM Corp., Armonk, NY, USA). Measurement data was expressed as mean ± standard deviation, the normal distribution and homogeneity of variance were compared by non-paired *t*-test between two groups and one-way analysis of variance (ANOVA) or repeated measurement ANOVA among multiple groups, followed by Turkey’s post-hoc test. Kaplan-Meier method was used to calculate the survival rate of the patients. Log-rank test was used for univariate analysis. Pearson correlation analysis was used to analyze the correlation of the observed indexes. A value of *p* < 0.05 was considered statistically significant.

## Results

### MALAT1 is highly expressed in GBC tissues and cells

**Fig. 1:**
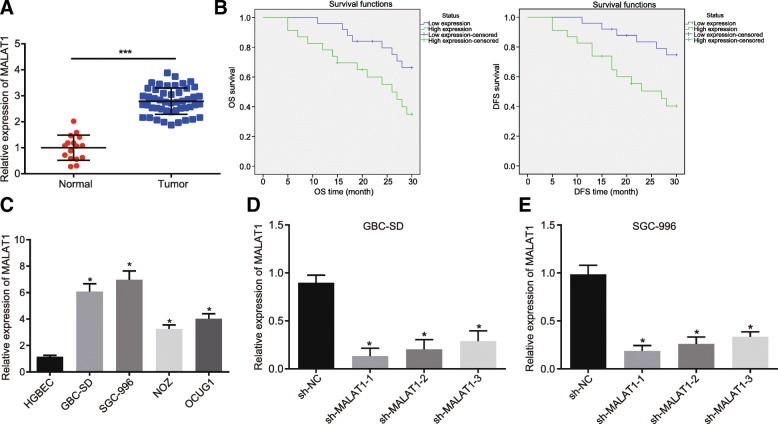
MALAT1 is up-regulated in GBC tissues and cells. **a**, the relative expression of MALAT1 in normal gallbladder tissues (*n* = 16) and GBC tissues (*n* = 48). * *p* < 0.05 vs. the normal gallbladder tissue. **b**, the correlation between the expression of MALAT1 and DFS and OS of patients analyzed by the Kaplan-Meier method. **c**, the relative expression of MALAT1 in GBC cells (GBC-SD, SGC-996, NOZ and OCUG1) and HGBECs determined by RT-qPCR. **d** and **e**, the relative expression of MALAT1 in GBC-SD and SGC-996 cells in the presence of MALAT1 silencing. * *p* < 0.05 vs. the HGBEC cell line or cells manipulated with sh-NC. The data was measurement data and expressed as mean value ± standard deviation. Comparison was analyzed by independent sample *t*-test. The experiment was repeated 3 times. MALAT1, metastasis associated lung adenocarcinoma transcript 1; GBC, gallbladder cancer; RT-qPCR, reverse transcription quantitative polymerase chain reaction; NC, negative control; HGBECs, human gallbladder epithelial cells; DFS, disease-free survival; OS, overall survival

Existing literature has identified ABI3BP as an anti-oncogene [13], however, the associated upstream regulatory mechanism is yet to be elucidated. MALAT1 has been demonstrated to promote the proliferation and metastasis of GBC [10]. In the present study, the content of MALAT1 in the GBC tissues (*n* = 48) as well as the normal gallbladder tissues (*n* = 16) was determined by RT-qPCR. The results of which revealed that the expression of MALAT1 in the GBC tissues was higher than that in the normal gallbladder tissues (*p* < 0.05, Fig. 1a). The correlation between the expression of MALAT1 and DFS as well as OS was evaluated using the Kaplan-Meier method. The results indicated that the OS (39.13%) of the patients with high expression of MALAT1 was significantly lower than that of patients exhibiting a low expression (68.00%, *p* = 0.026). An identical trend was identified in the DFS (43.48% vs. 76.00%, *p* = 0.012), highlighting the link between high expression of MALAT1 and the poor prognosis of GBC (Fig. 1b). Besides, in an attempt to investigate the effects of MALAT1 on the GBC cell lines, selected cell line was analyzed in subsequent experiments. The expression of MALAT1 in GBC-SD, SGC-996, NOZ, OCUG1 cells and HGBECs was evaluated by RT-qPCR. The results obtained revealed that the expression of MALAT1 in GBC-SD, SGC-996, NOZ, OCUG1 cells was significantly elevated when compared to the HGBECs (all *p* < 0.05). Furthermore, the GBC cell lines GBC-SD and SGC-996 exhibited the highest MALAT1 expression among the cell lines (*p* < 0.05). Therefore, the two GBC cell lines were selected for the subsequent experiments (Fig. 1c). The siRNA of MALAT1 was designed and the silence efficiency was assessed. The result obtained indicated that the expression of MALAT1 was down-regulated following treatment with sh-MALAT1–1, sh-MALAT1–2 and sh-MALAT1–3 in comparison to cells that had been transfected with sh-NC. Among them, the sh-MALAT1–2-treated cells contain the lowest expression of MALAT1 (*p* < 0.05). Hence, sh-MALAT1–2 was selected to deplete MALAT1 in the following experiments (Fig. 3d, e). The aforementioned results provided evidence suggesting that MALAT1 was highly expressed in GBC tissues and cells.

### MALAT1 silencing suppresses the proliferation, invasion and migration of GBC cells while contributing to senescence

**Fig. 2:**
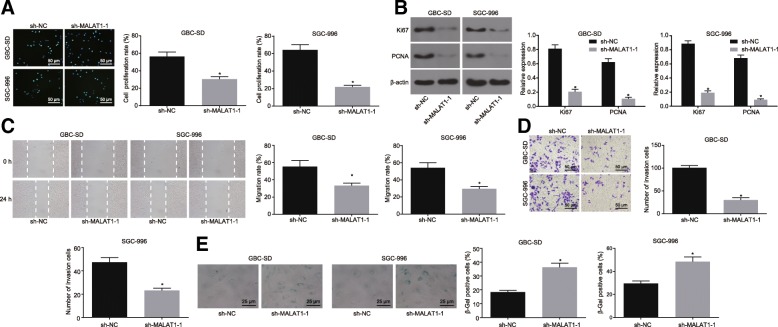
MALAT1 silencing restrains the proliferation, migration and invasion of GBC cells while promotes the apoptosis and senescence. **a**, the proliferation of GBC-SD and SGC-996 cells detected by EdU assay (200 ×). **b**, the protein expression of Ki67 and PCNA relative to β-actin in GBC-SD and SGC-996 cells determined by Western blot analysis. **c**, the migration of GBC-SD and SGC-996 cells detected by scratch test. **d**, the invasion of GBC-SD and SGC-996 cells detected by Transwell assay (200 ×). **e**, the senescence of GBC-SD and SGC-996 cells detected by SA-β-gal staining (400 ×). * *p* < 0.05 vs. the GBC-SD or SGC-996 cells transfected with sh-NC. The data was measurement data and expressed as mean value ± standard deviation. Comparison was analyzed by independent sample *t*-test. The experiment was repeated 3 times. MALAT1, metastasis associated lung adenocarcinoma transcript 1; GBC, gallbladder cancer; EdU, 5-ethynyl-2′-deoxyuridine; PCNA, proliferating cell nuclear antigen; SA-β-gal, senescence-associated β-galactosidase; NC, negative control

Our data further revealed that the expression pattern of MALAT1 in GBC tissues and cells, the focal point was put on the potential effects of MALAT1 on GBC cell biological functions, including cell viability by EdU assay, proliferation-related factors by western blot analysis, migration by scratch test, invasion by Transwell assay, and senescence by SA-β-gal staining. Our results indicated that when MALAT1 was silenced, the viability of the GBC-SD and SGC-996 cells was suppressed, and the expression of Ki67 and PCNA was reduced. Meanwhile, the capabilities of migration and invasion were inhibited, while the SA-β-gal activity was promoted (*p* < 0.05, Fig. 2a-e). The aforementioned findings suggested that MALAT1 silencing exerted the suppressive effect on the viability, proliferation, invasion and migration of GBC cells, but imposed a promoting effect on senescence.

### ABI3BP is poorly expressed in GBC tissues and cells

**Fig. 3:**
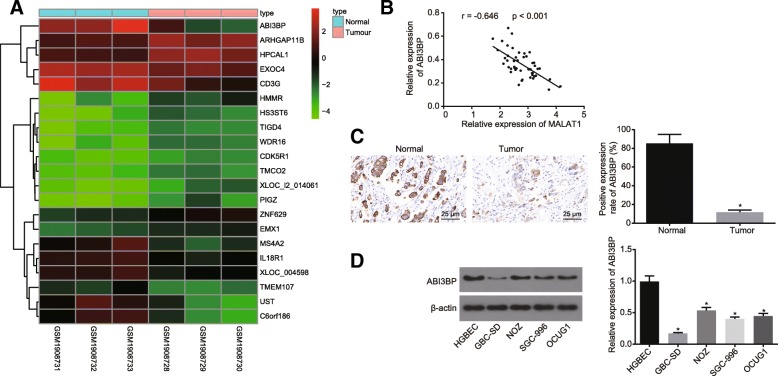
ABI3BP is poorly expressed in GBC tissues and cells. **a**, microarray data analysis of GSE74048 dataset. The abscissa indicates the sample type and the ordinate indicates the differential genes. Histogram on the upper right represents color gradation and rectangle refers to the expression level. **b**, the correlation analysis between expression of MALAT1 and ABI3BP in GBC tissues; **c**, the relative expression of ABI3BP in normal gallbladder tissues (n = 16) and GBC tissues (n = 48) detected by immunohistochemistry (400 ×). **d**, the relative expression of ABI3BP in the GBC cells (GBC-SD, SGC-996, NOZ and OCUG1) and HGBECs. * *p* < 0.05 vs. the normal gallbladder tissue or HGBEC cell line. The data was measurement data and expressed as mean value ± standard deviation. Comparison was analyzed by independent sample *t*-test between two groups and by one-way analysis of variance among multiple groups, followed by Turkey’s post-hoc test. The experiment was repeated 3 times. ABI3BP, ABI family member 3 binding protein; GBC, gallbladder cancer; HGBECs, human gallbladder epithelial cells

The expression profile of ABI3BP in GBC was investigated. The microarray data analysis of the GSE74048 datasets revealed that ABI3BP was poorly expressed in GBC (Fig. 3a). In order to figure out the correlation between the expression of MALAT1 and ABI3BP in the samples of GBC, we carried out the correlation analysis and found that the expression of MALAT1 and ABI3BP was negatively correlated (Fig. 3b). Meanwhile, the expression of ABI3BP was detected in the GBC tissues (*n* = 48) and the normal gallbladder tissues (*n* = 16) by immunohistochemical staining. The results indicated that compared with normal gallbladder tissue, the expression of ABI3BP was notably diminished among the GBC tissues (*p* < 0.05, Fig. 3c). The contents of ABI3BP in the GBC cells (GBC-SD, SGC-996, NOZ and OCUG1) and HGBECs were determined by Western blot analysis. The results revealed that the expression of ABI3BP in the GBC cells was considerably lower than that in the HGBECs, and the expression of ABI3BP in GBC-SD cells was the lowest (*p* < 0.05, Fig. 3d). Hence, the GBC cell line GBC-SD was selected for the subsequent experiments. The above findings provided evidence suggesting that the expression of ABI3BP was low in GBC tissues and cells.

### Restored ABI3BP inhibits GBC cell proliferation and migration while promoting senescence

**Fig. 4:**
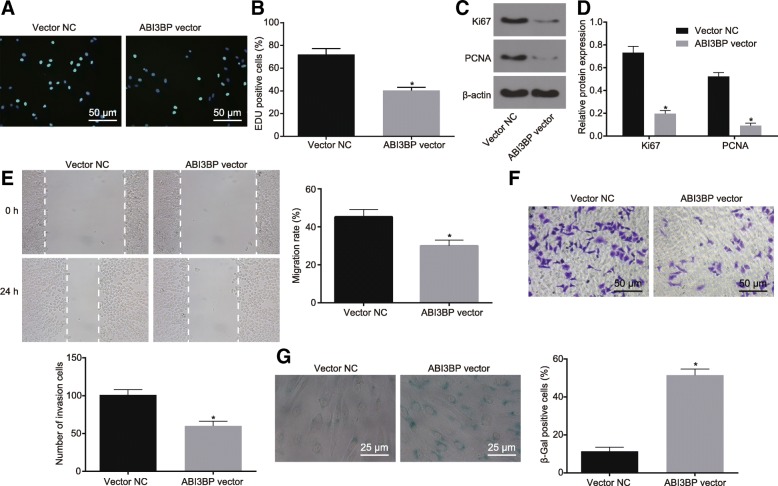
ABI3BP over-expression restrains the proliferation and metastasis of GBC cells while contributes to senescence. **a** and **b**, the proliferation of GBC-SD cells detected by EdU assay (200 ×). **c** and **d**, the protein expression of Ki67 and PCNA relative to β-actin in GBC-SD cells determined by Western blot analysis. **e**, the migration of GBC-SD cells detected by scratch test. **f**, the invasion of GBC-SD cells detected by Transwell assay (200 ×). **g**, the senescence of GBC-SD cells detected by SA-β-gal staining (400 ×). * *p* < 0.05 vs. the GBC cells manipulated with vector-NC. The data was measurement data and expressed as mean value ± standard deviation. Comparison was analyzed by independent sample *t*-test. The experiment was repeated 3 times. ABI3BP, ABI family member 3 binding protein; GBC, gallbladder cancer; EdU, 5-ethynyl-2′-deoxyuridine; PCNA, proliferating cell nuclear antigen; SA-β-gal, senescence-associated β-galactosidase; NC, negative control

With the results identified the ectopic expression of ABI3BP in GBC tissues and cells, the focus was shifted to the effects of ABI3BP on GBC cell viability using EdU assay, cell proliferation-related factors using western blot analysis, cell migration using scratch test, cell invasion using Transwell assay and cell senescence using SA-β-gal staining. The results demonstrated that compared with cells manipulated with vector-NC, cells manipulated with ABI3BP-vector displayed markedly enhanced ability of cell viability, weakened abilities of migration and invasion and promoted SA-β-gal activity, as well as significantly down-regulated expression of Ki67 and PCNA (*p* < 0.05, Fig. 4a-h). Taken together, the obtained results highlighted the suppressive effects of elevated ABI3BP expression on GBC cell viability, proliferation, migration and invasion while contributing to cell senescence.

### MALAT1 inhibits ABI3BP expression by recruiting EZH2 to ABI3BP promoter region

**Fig. 5:**
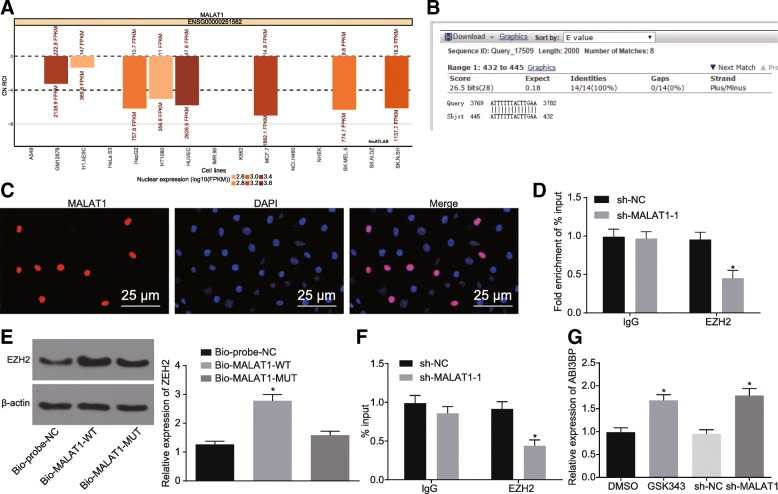
MALAT1 down-regulates the expression of ABI3BP through EZH2 recruitment in the promoter region of ABI3BP. **a**, the subcellular localization of MALAT1 predicted on lncATLAS website. **b**, Blast comparison between sequences of MALAT1 and ABI3BP promoter. **c**, the subcellular localization of MALAT1 detected by FISH assay (400 ×). **d**, the enrichment of EZH2 by MALAT1 detected by RIP assay. **e**, the relative expression of EZH2 relative to β-actin detected by RNA pull down. **f**, EZH2 enrichment in the ABI3BP promoter region detected by ChIP assay. **g**, the relative expression of ABI3BP determined by RT-qPCR. * *p* < 0.05 vs. the cells treated with sh-NC, Bio-probe-NC or DMSO. The data was measurement data and expressed as mean value ± standard deviation. Comparison was analyzed by independent sample *t*-test between two groups and by one-way analysis of variance among multiple groups, followed by Turkey’s post-hoc test. The experiment was repeated 3 times. MALAT1, metastasis associated lung adenocarcinoma transcript 1; ABI3BP, ABI family member 3 binding protein; EZH2, enhancer of zeste homolog 2; FISH, fluorescence in situ hybridization; RIP, RNA immunoprecipitation; ChIP, chromatin immunoprecipitation; RT-qPCR, reverse transcription quantitative polymerase chain reaction; NC, negative control; DMSO, dimethyl sulfoxide

EZH2 was identified to be related to 3′-end of MALAT1 and inhibit expression of upstream genes [17]. As predicated by the lncATLAS website (http://lncatlas.crg.eu/) MALAT1 was mainly localized in the nucleus, which was further confirmed by the results of the FISH assay (Fig. 5a, c). Blast comparison results revealed that the complementary binding between MALAT1 and ABI3BP promoters might exist in the form of RNA-DNA (Fig. 5b). It was suggested that MALAT1 may inhibit the expression of ABI3BP by recruiting EZH2 to ABI3BP promoter region. The enrichment of EZH2 by MALAT1 was assessed by RIP and the result revealed that the enrichment of EZH2 diminished significantly in cells where MALAT1 was silenced (*p* < 0.05, Fig. 5d). The binding of MALAT1 to EZH2 was further verified by RNA pull down. The results demonstrated that Bio-MALAT1-WT pull-down more EZH2 relative to that of the Bio-probe and Bio-MALAT1-MUT, suggesting that MALAT1 promoted the enrichment of EZH2 (*p* < 0.05, Fig. 5e). The enrichment of methyltransferase EZH2 in the ABI3BP promoter region was detected by ChIP assay, the results of which revealed that sh-EZH2 treatment had a less significant enrichment than sh-NC treatment (*p* < 0.05, Fig. 5f). The expression of ABI3BP in the cells that were treated with sh-MALAT1 or EZH2 inhibitor (GSK343) was subsequently determined, the results of which revealed that the expression of ABI3BP increased in cells treated with GSK343 in comparison to treatment with DMSO (*p* < 0.05). Higher expression of ABI3BP was identified among cells treated with sh-MALAT1 than that in sh-NC (*p* < 0.05, Fig. 5g). Taken together, a conclusion was drawn that MALAT1 inhibited the expression of ABI3BP by recruiting EZH2 to the promoter region of ABI3BP.

### EZH2 inhibits ABI3BP expression by driving H3K27 methylation

**Fig. 6:**
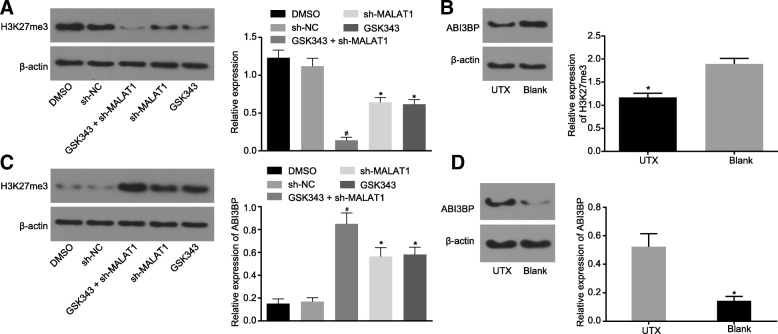
EZH2 down-regulates the expression of ABI3BP via H3K27 methylation. **a**, the relative expression of H3K27me3 determined by Western blot analysis following the treatment of GSK343 (EZH2 inhibitor) or sh-MALAT1. **b**, the relative expression of H3K27me3 determined by Western blot analysis following the treatment of UTX (histone demethylase). **c**, the relative expression of ABI3BP determined by Western blot analysis following the treatment of GSK343 (EZH2 inhibitor) or sh-MALAT1. **d**, the relative expression of ABI3BP determined by Western blot analysis following the treatment of UTX (histone demethylase). * *p* < 0.05 vs. the cells treated with sh-NC or DMSO. # *p* < 0.05 vs. the cells transfected with sh-MALAT1. The data was measurement data and expressed as mean value ± standard deviation. Comparison was analyzed by independent sample *t*-test between two groups and by one-way analysis of variance among multiple groups, followed by Turkey’s post-hoc test. The experiment was repeated 3 times. EZH2, enhancer of zeste homolog 2; ABI3BP, ABI family member 3 binding protein; H3K27me3, trimethylation of lysine 27 on histone H3 protein subunit; MALAT1, metastasis associated lung adenocarcinoma transcript 1; UTX, ubiquitously transcribed tetratricopeptide repeat on chromosome X; NC, negative control; DMSO, dimethyl sulfoxide

The level of H3K27me3 methylation was investigated by Western blot analysis in order to further verify the relationship between MALAT1, EZH2 and ABI3BP. The results revealed that compared to the sh-NC group, the level of H3K27me3 methylation was decreased in the sh-MALAT1 group. Meanwhile, the level of H3K27me3 methylation in cells treated with GSK343 was significantly lower than that in cells treated with DMSO. The combination of treatment with GSK343 + sh-MALAT1 enhanced the down-regulation of H3K27me3 methylation (*p* < 0.05, Fig. 6a). The effect of histone demethylase on H3K27 methylation was evaluated by determining the expression of H3K27me3 after UTX treatment. The result revealed that the expression of H3K27me3 was remarkably decreased after UTX treatment (*p* < 0.05, Fig. 6b). The expression of ABI3BP was again determined by western blot analysis to explore the relationship among MALAT1, EZH2 and ABI3BP. Compared with cells treated with sh-NC, sh-MALAT1 treatment exhibited a significant increase in the expression of ABI3BP. GSK343 treatment was found to remarkably up-regulate the expression of ABI3BP compared to the DMSO treatment. The same changing tendency was also induced by GSK343 + sh-MALAT1 (*p* < 0.05, Fig. 6c). Further attempts were made to investigate the effects of histone demethylase on ABI3BP expression by western blot analysis after UTX treatment. The results showed that UTX treatment significantly up-regulated the expression of ABI3BP (*p* < 0.05, Fig. 6d). To sum up, these findings proved that MALAT1 silencing or histone methylation suppressing promoted the expression of ABI3BP.

### MALAT1 silencing restrains the development of GBC cells by promoting ABI3BP expression

**Fig. 7:**
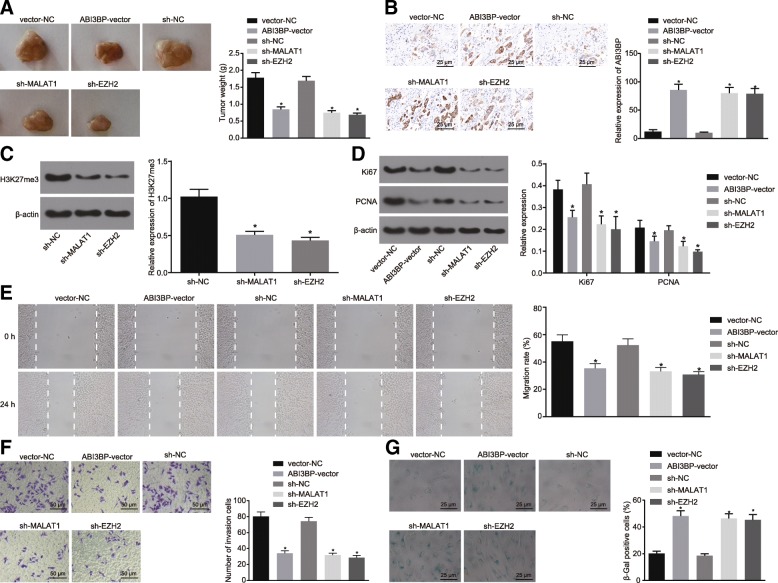
Over-expressed ABI3BP and silenced MALAT1 and EZH2 impede GBC cell proliferation, migration, invasion and tumorigenic potential while accelerate senescence. **a**, representative images of resected tumors from nude mice and tumor weights. **b**, the relative expression of ABI3BP determined by immunohistochemistry (400 ×). **c**, the relative expression of H3K27me3 relative to β-actin determined by Western blot analysis. **d**, the relative expression of Ki67 and PCNA relative to β-actin determined by Western blot analysis. **e**, the cell migration detected by scratch test. **f**, the cell invasion detected by Transwell assay (200 ×). **g**, the cell senescence detected by SA-β-gal staining (400 ×). * *p* < 0.05 vs. nude mice treated with vector-NC or sh-NC. The data was measurement data and expressed as mean value ± standard deviation. Comparison was analyzed by one-way analysis of variance among multiple groups, followed by Turkey’s post-hoc test. The experiment was repeated 3 times. ABI3BP, ABI family member 3 binding protein; MALAT1, metastasis associated lung adenocarcinoma transcript 1; EZH2, enhancer of zeste homolog 2; GBC, gallbladder cancer; H3K27me3, trimethylation of lysine 27 on histone H3 protein subunit; PCNA, proliferating cell nuclear antigen; SA-β-gal, senescence-associated β-galactosidase; NC, negative control

The roles of MALAT1, EZH2 and ABI3BP in the development of GBC were further explored through xenograft tumor in nude mice. The ABI3BP-vector, sh-MALAT1, sh-EZH2, vector-NC and sh-NC were delivered respectively into the GBC-SD cells, which were then inoculated into the nude mice. The tumor size and weight were measured accordingly. The results obtained revealed that the tumor size in the mice treated with over-expressed ABI3BP was smaller than that in the vector-NC group (*p* < 0.05). Meanwhile, the tumors in the mice treated with sh-MALAT1 or sh-EZH2 was smaller than that treated with sh-NC (*p* < 0.05, Fig. 7a). The expression of ABI3BP was elevated in the ABI3BP-vector group in comparison with the vector-NC group, while enhanced in the sh-MALAT1 and sh-EZH2 groups when compared to the sh-NC group (*p* < 0.05, Fig. 7b), while the H3K27me3 methylation exhibited diminished levels among the mice treated with sh-MALAT1 and sh-EZH2 (*p* < 0.05, Fig. 7c). The effects of ABI3BP, MALAT1 and EZH2 on the proliferation, invasion, migration and senescence of GBC cells were evaluated, the results of which revealed that after the elevation of ABI3BP, the expression of Ki67 and PCNA in tumors were reduced, while the cell migration and invasion abilities were diminished, along with stimulated SA-β-gal activity. The same effects were observed among the mice treated with sh-MALAT1 and sh-EZH2 (*p* < 0.05, Fig. 7d-h). The aforementioned findings suggested that ABI3BP over-expression, MALAT1 or EZH2 silencing suppressed GBC proliferation while promoting cell senescence.

### Silencing of ABI3BP rescues MALAT1 silencing-induced inhibition of GBC cell proliferation, invasion, migration and tumorigenic potential

**Fig. 8:**
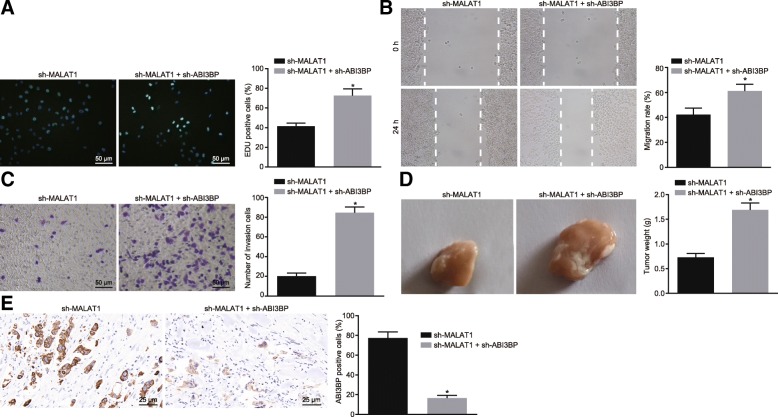
Suppression of proliferation, invasion, migration and tumorigenic potential induced by MALAT1 silencing could be rescued by ABI3BP silencing. **a**, the proliferation determined by EdU assay (200 ×). **b**, the cell migration detected by scratch test. **c**, the cell invasion detected by Transwell assay (200 ×). **d**, representative images of resected tumors from nude mice and tumor weights. **e**, the relative expression of ABI3BP determined by immunohistochemistry (400 ×). * *p* < 0.05 vs. cells or nude mice treated with sh-MALAT1. The data was measurement data and expressed as mean value ± standard deviation. Comparison was analyzed by independent sample *t*-test between two groups. The experiment was repeated 3 times

EdU assay, scratch test, and Transwell assay were employed to investigate the effect of the interference treatment of both MALAT1 and ABI3BP on the viability, migration and invasion of GBC cells. The results demonstrated that the addition of sh-ABI3BP could reverse the inhibitory role of sh-MALAT1 on GBC cell proliferation, migration and invasion (*p* < 0.05, Fig. 8a-c). The GBC-SD cells transfected with the sh-MALAT1 alone or with sh-ABI3BP vector were inoculated in nude mice, with the tumor volume and weight of the nude mice analyzed and compared accordingly. The results revealed that the tumor volume and weight in the sh-MALAT1 + sh-ABI3BP-treated mice was significantly elevated when compared with the sh-MALAT1-treated mice (*p* < 0.05, Fig. 8d). Immunohistochemistry were subsequently was used to detect the expression of ABI3BP in tumor tissues of each group. The results revealed that the expression of ABI3BP in the sh-MALAT1 + sh-ABI3BP group was significantly lower than that in the sh-MALAT1 group (*p* < 0.05, Fig. 8e). This indicated that inhibiting the expression of MALAT1 and ABI3BP at the same time could promote the proliferation, invasion and migration of GBC cells, as well as tumor formation.

## Discussion

GBC represents an aggressive tumor that is often accompanied by poor prognosis even after the chemo- and/or radio-therapy. Consequently, the underlying molecular mechanisms associated with the malignancy require further investigation to identify more effective therapeutic methods [18]. The notable involvement of various lncRNAs in GBC has been reported, in which MALAT1 is mentioned to harbor oncogenic properties in GBC [19]. The current study suggested that MALAT1 silencing could serve as a promising treatment strategy by suppressing the GBC growth and accelerating cell senescence. Our results provided verification suggesting the inhibition of the progression of GBC due to the elevated ABI3BP through reduction of EZH2 recruitment and H3K27 methylation.

Initially, the expression profiles of MALAT1 and ABI3BP in GBC tissues and cells were investigated, the results demonstrated that MALAT1 was abundantly expressed while the expression of ABI3BP down-regulated when compared with normal gallbladder tissues and cells. Consistent with the observations of our study, a previous report concluded that MALAT1 is highly expressed in GBC tissue while GBC cell proliferation is inhibited along with stimulated cell apoptosis when siRNA against MALAT1 is stably introduced into GBC cells [20]. Elevated MALAT1 expression has been reported to be positively correlated to lymphatic metastasis and tumor size but negatively correlated with the overall survival of GBC patients [21]. A follow-up analysis asserted that MALAT1 could function as a potential biological indicator of GBC in relation to recurrence, metastasis and prognosis of patients suffering from GBC [22]. Consistently, in vitro and in vivo experiments adduced available evidence demonstrated the diagnostic value of silencing MALAT1 exemplified by the suppression of GBC cell growth and metastatic characteristics along with potentiated senescence when sh-MALAT1 was introduced. Additionally, a key finding in our study revealed that elevated ABI3BP expression could significantly inhibit GBC cell proliferation and metastasis while increasing cell population senescence. Studies have attested the anti-oncogenic role of ABI3BP indicating that the ectopic expression of ABI3BP could weaken cancer cell growth properties and induce senescence [13]. The involvement of ABI3BP in tumorigenesis has suggested that ABI3BP depletion is capable of inducing oncogenic transformation [23]. ABI3BP restoration has been highlighted as a significant factor in relation to thyroid tumors, whereby tumor growth and invasion are impeded while cell senescence is promoted [24]. Besides, the expression of ABI3BP has been reported to undergo decreases in lung cancer cell lines, suggesting its function as a biomarker for lung cancer progression [25].

Our results indicated that ABI3BP could be down-regulated by MALAT1 through the recruitment of EZH2 binding to the ABI3BP promoter region by H3K27 methylation. Importantly, EZH2 has been reported to be related to 3′-end of MALAT1 and down-regulate the expression of E-cadherin in colorectal cancer [17]. Moreover, MALAT1 has been shown to bind to EZH2, resulting in the inhibition of the tumor suppressor protocadherin 10 contributing to gastric cell migration and invasion [26]. EZH2 is widely understood to be a histone-lysine N-methyltransferase enzyme involving DNA methylation in association with the development of GBC [27]. As a subunit of polycomb repressive complex 2 (PRC2), EZH2 possesses the ability to recruit DNA methyltransferases to the target promoter, resulting in gene silencing and DNA methylation [28]. PRC2 has been recognized in various studies as a mediator of H3K27me3, which may repress transcription and result in chromatin condensation [29]. Furthermore, EZH2 has also been reported to target H3K27 as well as the only methyltransferase of H3K27me3 [30]. EZH2-mediated H3K27me3 can induce epigenetic silencing of microRNA-128, further causing carcinogenesis of cigarette smoke [31]. In our study, it was proved as well that EZH2-mediated H3K27me3 down-regulated ABI3BP, thereby contributing to the progression of GBC.

## Conclusion

**Fig. 9:**
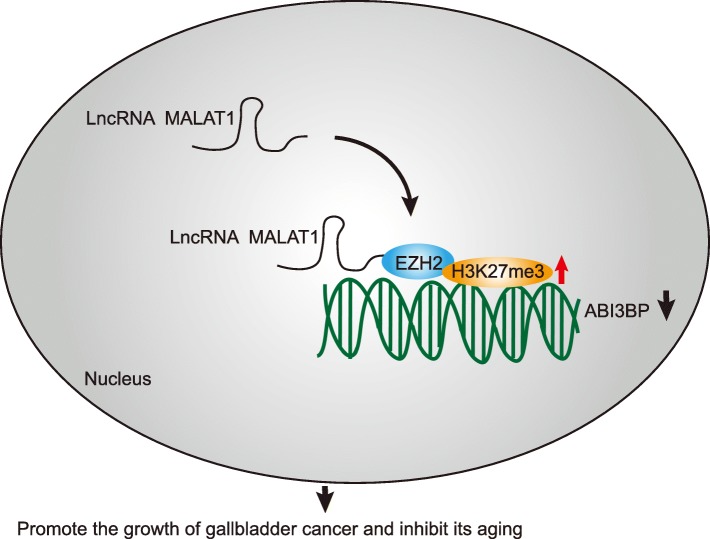
The mechanism of MALAT1 on GBC. MALAT1 silencing or ABI3BP restoration can facilitate the treatment of patients with GBC by exerting suppressive effects on GBC cell growth properties both in vitro and in vivo accompanied by strengthened the ability of cell senescence

Taken together, the key findings of the current study suggest that MALAT1 silencing or ABI3BP restoration can exert suppressive effects on GBC cell growth both in vitro and in vivo accompanied by strengthened cell senescence (Fig. 9). Furthermore, the discovery may provide significance for the intervention and treatment of GBC. An additional study using a large cohort of samples from patients with GBC is required to confirm the prognostic value of MALAT1 in GBC treatment.

## Data Availability

The datasets generated and/or analysed during the current study are available from the corresponding author on reasonable request.

## Abbreviations

BCA: Bicinchoninic acid
BSA: Bovine serum albumin
ChIP: Chromatin immunoprecipitation
DAB: Diaminobenzidine
DMEM: Dulbecco’s Modified Eagle Medium
DMSO: Dimethyl sulfoxide
ECL: Enhanced chemiluminescence
EZH2: Enhancer of zeste homolog 2
FBS: Fetal bovine serum
FISH: Fluorescence in situ hybridization
FITC: Fluorescein isothiocyanate
GAPDH: Glyceraldehyde 3-phosphate dehydrogenase
GBC: Gallbladder cancer
HRP: Horseradish peroxidase
IgG: Immunoglobulin G
IHC: Immunohistochemistry
lncRNAs: Long non-coding RNAs
MALAT1: Metastasis associated lung adenocarcinoma transcript 1
MUT: Mutant
PCNA: Proliferating cell nuclear antigen
PI: Propidium iodide
PVDF: Polyvinylidene fluoride
RIP: RNA immunoprecipitation
RIPA: Radioimmunoprecipitation assay
RPMI: Roswell Park Memorial Institute
RT-qPCR: Reverse transcription quantitative polymerase chain reaction
SA-β-gal: Senescence-associated-β-galactosidase
SDS-PAGE: Sodium dodecyl sulfate-polyacrylamide gel electrophoresis
shRNAs: Specific short hairpin;RNAs
WT: Wild type
